# An inexpensive fast memory module for rapid acquisition of digital data

**DOI:** 10.1155/S1463924685000153

**Published:** 1985

**Authors:** David T. Rossi, Harry L. Pardue

**Affiliations:** Department of Chemistry Purdue University West Lafayette Indiana 47907 USA


Journal of Automatic Chemistry/*Journal of Clinical Laboratory Automation*, Vol. 7, No. 2 (Apr–Jun 1985), pp. 64–68

### An inexpensive fast memory module for rapid acquisition of digital data

**David T. Rossi and Harry L. Pardue***

*Department of Chemistry, Purdue University, West Lafayette, Indiana 47907, USA*

Spectroscopic applications of imaging systems have grown at prolific rates in recent years [1]. Unfortunately, because of the data intensiveness of these imaging detectors, their utility is sometimes limited by the ability of host data systems to collect and process images. Although it is usually possible to expand the size and speed of a data system to accommodate the increased rates and volume for such data sets, the complexity and cost may not always be justified.

This paper describes a simple, inexpensive approach to the problem of data collection for a diode array spectrometer. The data system is centred around a memory module which is peripheral to a host computer. The memory module involves 64 kilobytes of dynamic random access memory (RAM), controlled by a recently introduced dynamic RAM controller/driver. With this memory system it is possible to equal the data collection performance of substantially more complex data systems at a fraction of the cost. Although the data system described here has been used in conjuction with a diode array spectrometer, the design is sufficiently general that it can be applied in most circumstances demanding rapid collection of predigitized data.

To evaluate some of the capabilities of this data system, the diode array spectrometer was applied to temporal monitoring of a mercury pen lamp and the kinetic monitoring of an electrochemically initiated reaction by a thin-layer technique [2]. The selected test system involves the irreversible hydrolysis of p-benzoquinoneimine (QI), electrochemically generated from p-aminophenol (PAP), to form p-benzoquinone (Q) [3].

### Experimental section

### *Data system*

Figure 1 is a block diagram of the electronic data system for the diode array spectrometer. The optical system has been described previously [4]. The detector is a solid state photodiode array (Model 1412, Princeton Applied Research, Princeton, New Jersey, USA) containing 1024 pixels. Scan parameters, such as integration and scan time, as well as data digitization, are handled by the detector-controller (Model 1218, Princeton Applied Research). Detector scanning is initiated by the host computer (PDP 12-30, Digital Equipment Corporation, Maynard, Massachusetts, USA) through a general-purpose parallel interface, a network of encoding logic, and a control bus multiplexer, to the detector-controller. Alternatively, scanning can be initiated through a switch register to allow spectral scans to be synchronized with the initiation of experiments. The digitized data from the detector-controller are directed along the data bus, through a series of latches and drivers, to the memory module where they are stored. A flag pulse from the detector-controller strobes each datum into memory. The data rate during a scan is 39·2 kHz. A flag pulse, marking the end of a scan, is transmitted through the parallel interface to the computer. The computer therefore needs only to count the scans (40 Hz or less) and halt data collection when the desired number has been reached. The spectral data contained in the memory module is then transferred to the host computer memory, under program control, for processing, bulk storage, and/or read-out.

A detailed schematic diagram for the memory module is shown in figure 2. Data from the instrument are sent through the data line receiver/drivers (ICs 19 and 34) to the dynamic RAM (ICs 0 to 11). Memory READ, WRITE and REFRESH functions are automtically accomplished by applying a small number of electronic signals to the RAM controller (IC 14, 74S409, Monolithic Memories Incorporated, Sunnyvale, California, USA). A detailed description of the controller operation is given elsewhere [5].

The WRITE cycle is selected when the host computer enables ICs 19 and 34. This is done by clearing control bit 10 of the parallel interface and setting control bit 8 and channel 15 simultaneously to strobe this information into a latch (IC 13). This procedure also selects the instrument flag line which is routed, through pulse shaping logic (IC 12) and through a multiplexer (IC 14), to address counters (ICs 22, 24–27), and timing circuitry (ICs 17, 32, 35, 36, 38). Figure 3 summarizes the timing for this mode of operation. As the address from ICs 22 and 24–27 becomes valid, the WRITE enable strobe, WIN, goes low. The data is written into memory 680 ns (tl) later by forcing the address strobe, ADS, the chip select for the memory controller, CS, and the row address strobe input, RASIN, low simultaneously.

Data are transferred from the memory module to the host computer in the READ cycle. This is accomplished by setting the parallel interface control bit 10, and strobing control bit 8 and channel 15 simultaneously. At this point, the address counters are reset to memory address FFFF hex, the data line receiver/drivers are disabled, and the computer flag line (parallel interface channel 17) is selected by the multiplexer (IC 14). The timing for this mode of operation is summarized in figure 4. To read the

* Corresponding author.

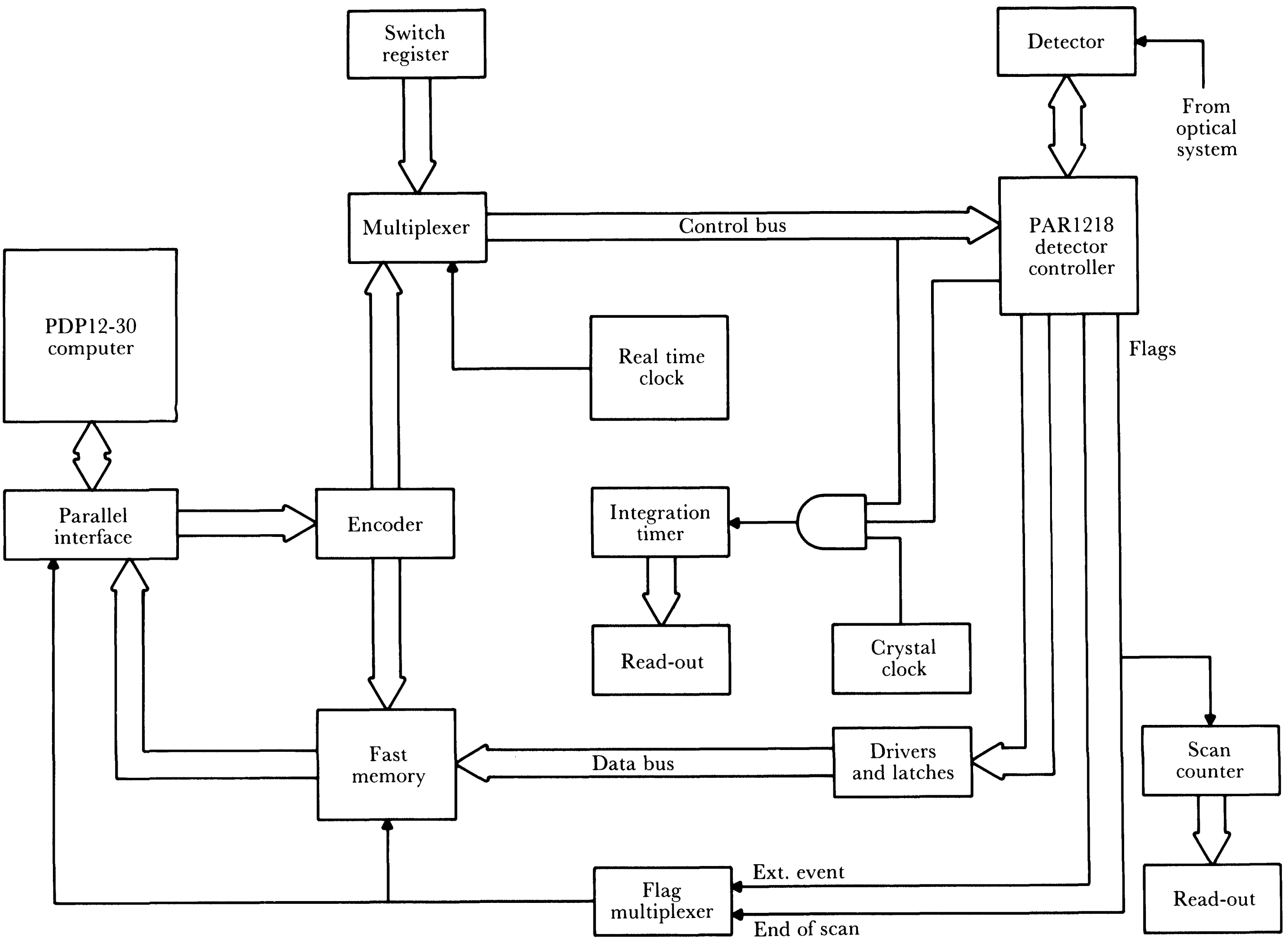

*Figure 1. Shematic representation of data acquisition system and computer/operator interface for diode array spectrometer.*

first data point a flag pulse from the host computer incriments the address to 0000 hex, ADS and CS go low together and, after a 400 ns (t8) delay, RASIN goes low. The datum appears on the bus, is strobed into a row of latches (ICs 18, 28 and 33), and is transmitted by open collector line drivers (ICs 30 and 31) to the parallel interface. After the host computer has successfully competed storing the datum in its own memory, it can receive the next point by simply applying a pulse to channel 17 of the parallel interface. This process is repeated until all data for an entire scan are in the host computer memory.

Figure 5 summarizes the timing in the refresh mode. Refreshing of the dynamic RAM is accomplished by applying a negative going CS pulse to the dynamic RAM controller when RASIN and the system clock, RFCK, are both high, at a time when neither READ nor WRITE processes are occurring. This synchronization is accomplished by using a common system clock for detector-controller and dynamic RAM controller. The circuitry provided by IC 16, 17, 20, 23, 29 and 37 in figure 2 serves to shape the clock pulse chain coming from the detector-controller and adjust additional timing parameters.

*Thin layer cell*

The thin layer cell was modelled after a design by Murray, Heineman and O'Dom [2]. An aqueous solution of PAP is irreversibly oxidized to QI by application of +0·904 V versus a saturated silver/silver chloride electrode.

*Reagents*

p-aminophenol (Aldrich, Milwaukee, Wisconsin, USA) and sulphuric acid (Mallinckrodt, Paris, KT) were used without further purification. The intermediate, QI, was electrochemically generated in the thin layer cell using a conventional electrolysis potentiostat [6].

### Results

Figure 6 demonstrates data collection at the fastest possible rate (as limited by the scan speed of the spectrometer) of 39·2 kHz with no delay between scans. This corresponds to one 1024 point spectrum taken every 25 ms. The data represent spectral intensity for a mercury pen lamp as a function of wavelength and time. Faster data rates should be possible by increasing the frequency of the sytem clock in the diode array spectrometer. Because the memory is synchronized to the system clock, some adjustments in memory timing would be necessary. Also, faster scanning necessarily implies a decrease in integration time and a subsequent decrease in the signal-to-noise ratio.

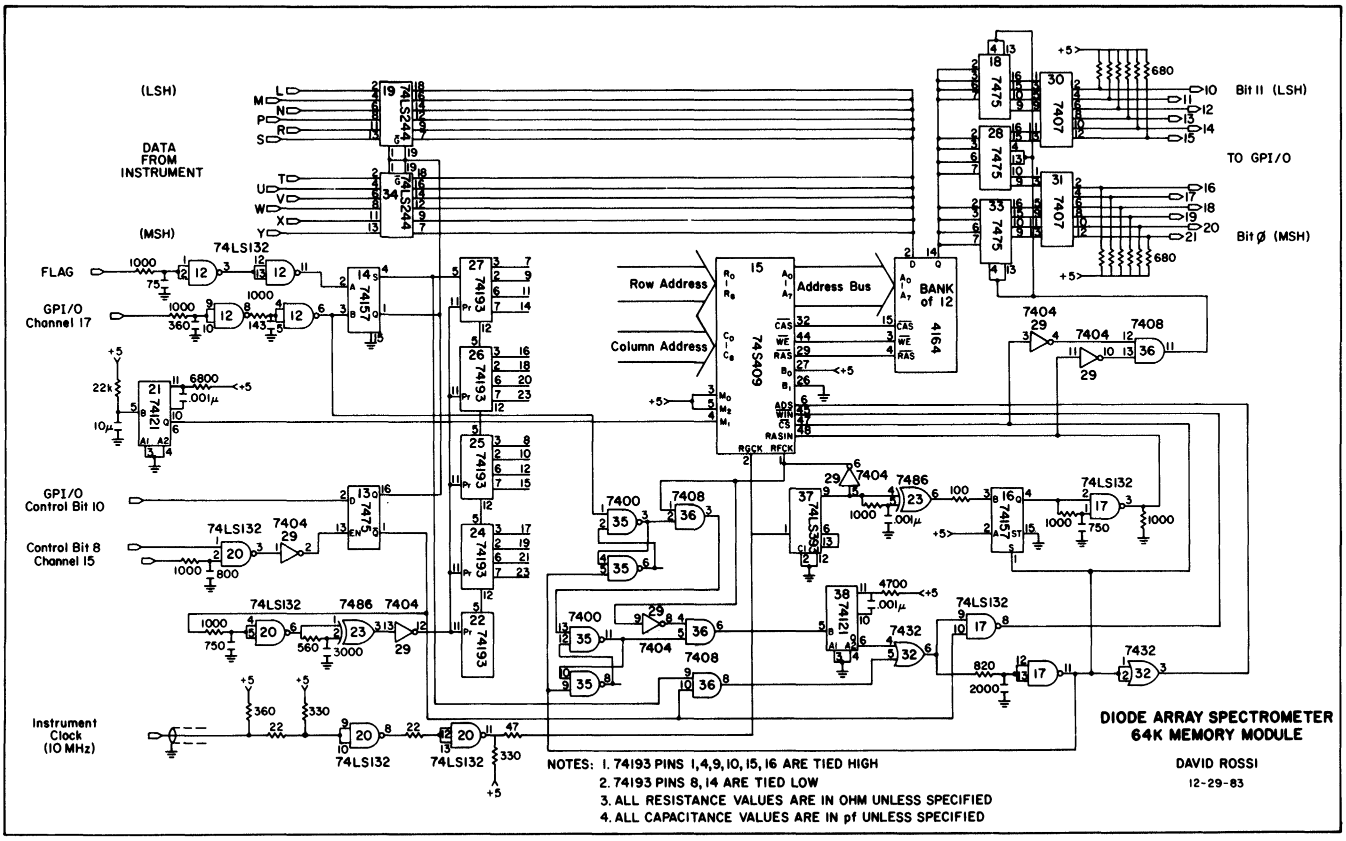

*Figure 2. Schematic drawing of 64 kilobyte fast memory module for diode array spectrometer.*

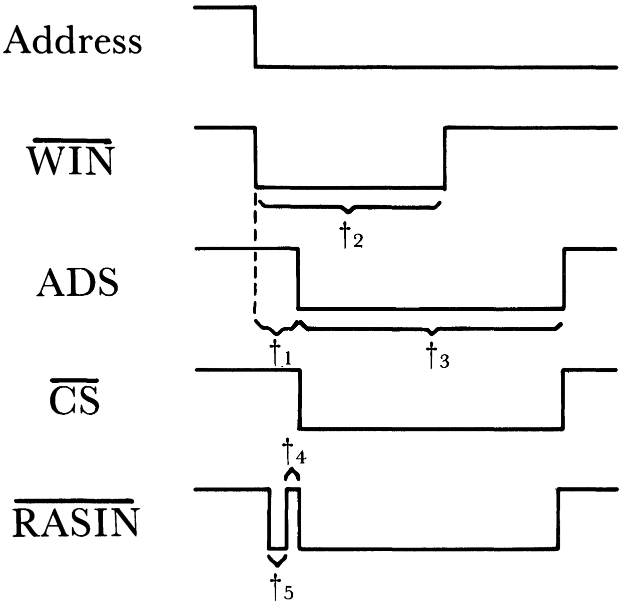

*Figure 3. Timing diagram for memory access/write cycle. t1, 680 ns; t2, 3·1 µs; t3, 4·4 µs; t4, 200 ns; t5, 270 ns.*

The optically transparent thin layer electrochemical cell initially contained an aqueous solution of 9·3 × $10^{-3}$ M p-aminophenol in 0·90 M sulphuric acid. As the potential is applied to the cell, PAP is irreversibly oxidized to QI. Figure 7 shows the increse in absorbance as the oxidation proceeds. A shift in absorption maximum from 270 to 250 nm occurs as absorbance for QI begins to predominate that for the PAP species.

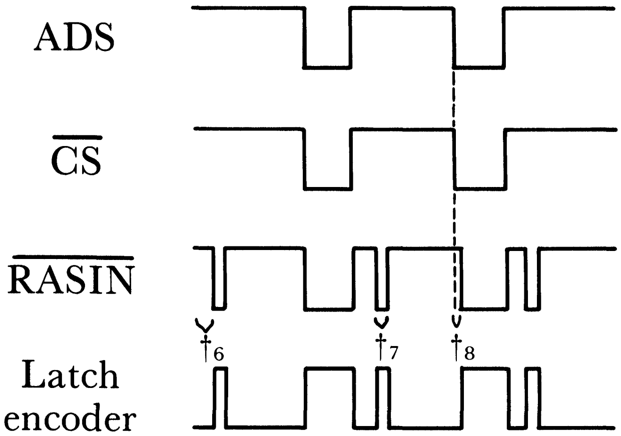

*Figure 4. Timing diagram for memory access/read cycle. t6, 1·7 µs; t7, 400 ns; t8 400 ns.*

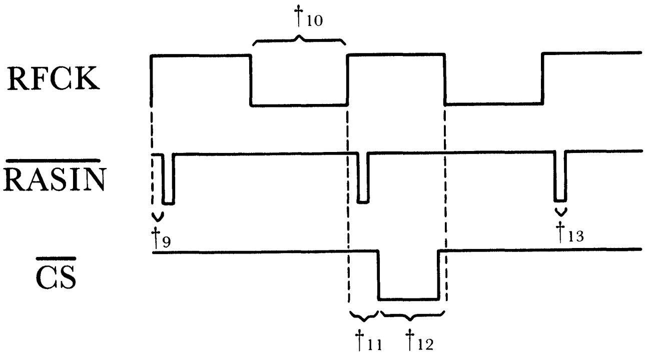

*Figure 5. Timing diagram for memory refresh function. t9, 480 ns; t10, 6·1 µs; t11, 2·5 µs; t12, 4·4 µs; t13, 450 ns.*

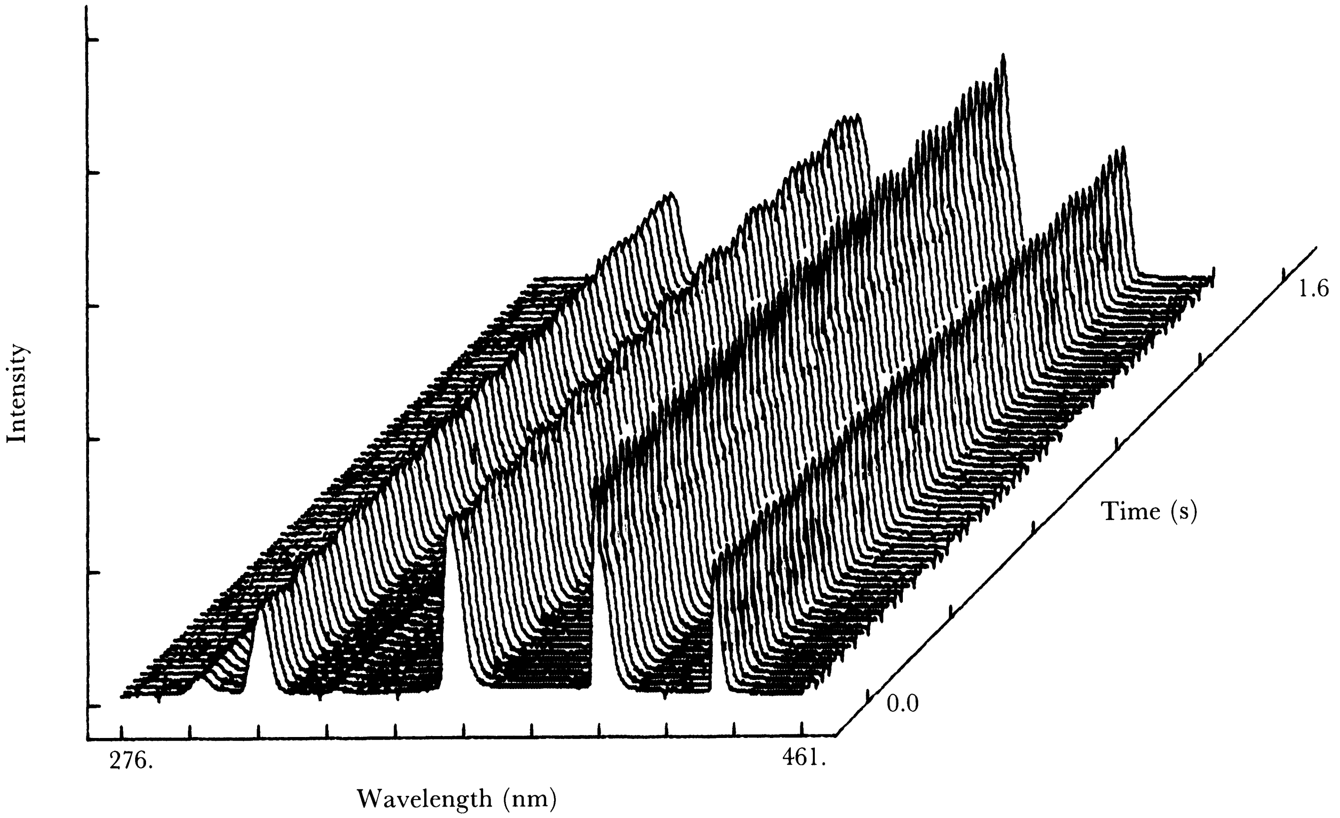

*Figure 6. Intensity versus wavelength and time for a mercury pen lamp emission.*

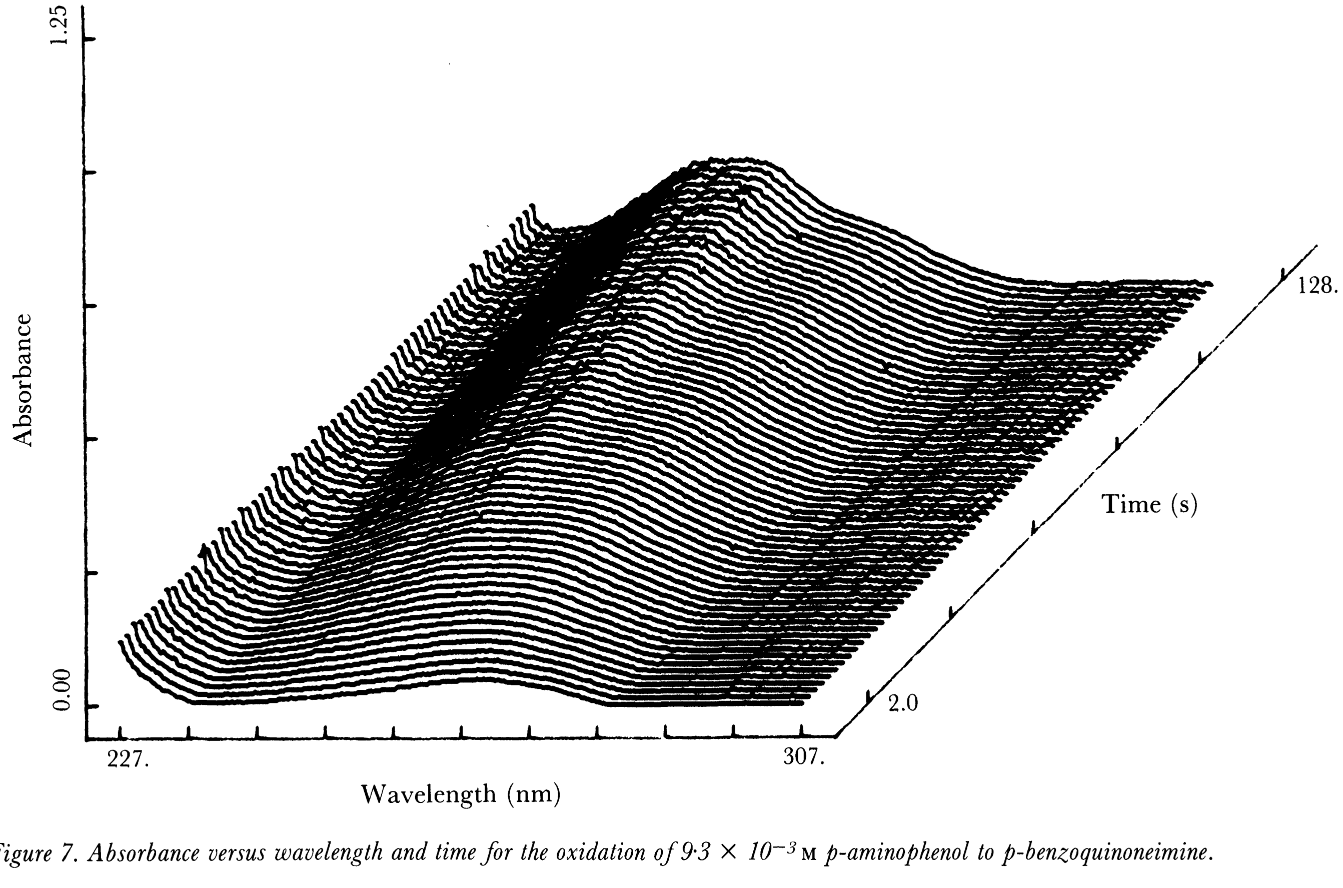

*Figure 7. Absorbance versus wavelength and time for the oxidation of 9·3 × $10^{-3}$ M p-aminophenol to p-benzoquinoneimine.*

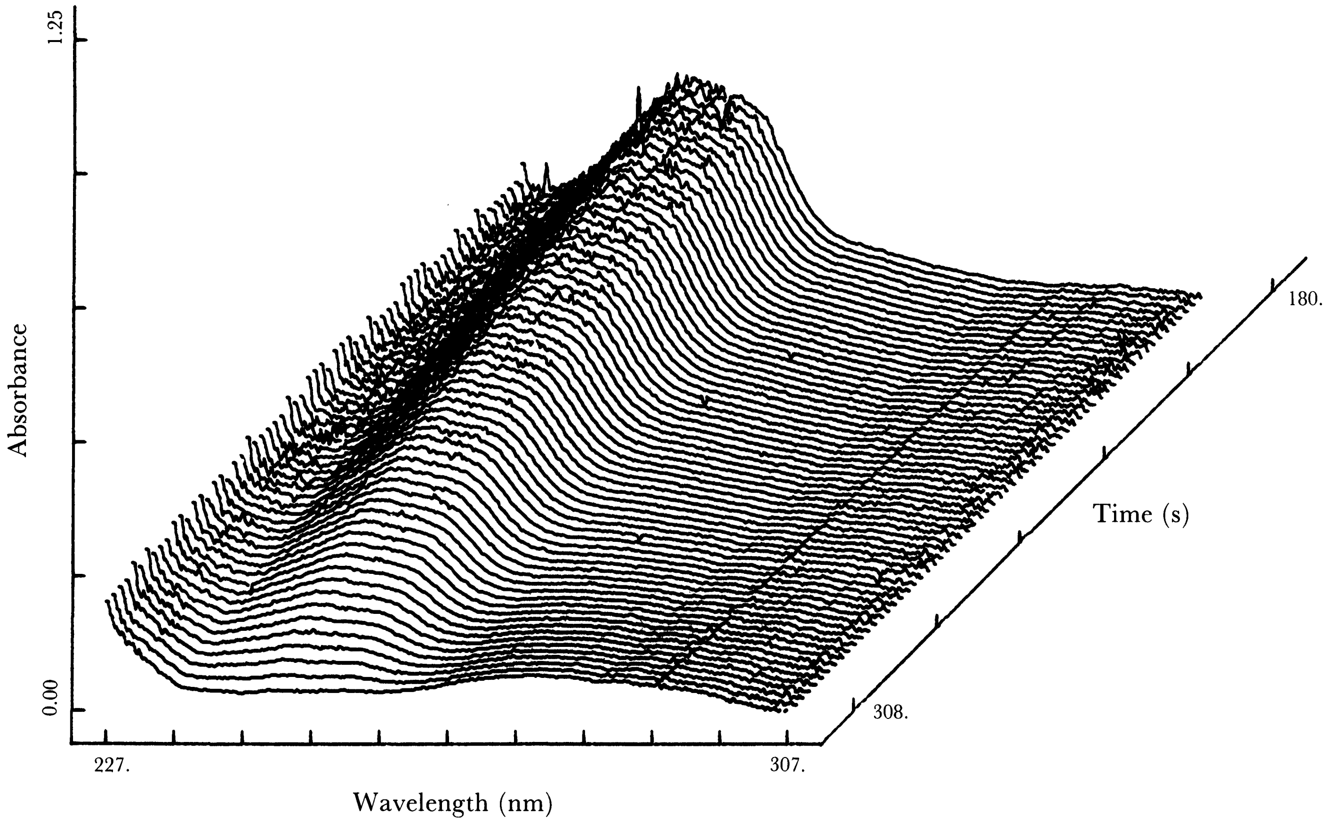

*Figure 8. Absorbance versus wavelength and time for the decay of p-benzoquinoneimine, electrogenerated in the thin layer cell from 9 3 × $10^{-3}$* M *p-aminophenol, to form p-benzoquinone.*

After approximately 3 min at these conditions, the oxidiation of PAP to QI is essentially complete and the hydrolysis of QI to Q proceeds. Figure 8 displays absorbance as a function of wavelength and time for the hydrolysis. Because the rate of hydrolysis is much slower than the rate of oxidation, the spectral changes due to the oxidation can be temporarily distinguished from those of the hydrolysis. This is not always true, however, depending on the cell design and the chemical system [3]. It is in these situations that rapid scanning spectroscopy and multiwavelength data-processing techniques may prove useful for deconvolution of the complex kinetic data.

### Discussion

Some commercially available diode array instruments, for both spectroscopic and chromatographic applications, suffer from very limited temporal and spectral resolution, largely as a result of restrictions in data collection efficiency. These performance restrictions, to a large degree, dictate the scope of applications for these diode array systems. The memory module approach offers a way to substantially speed up data collection.

The memory module can be useful in a variety of stiuations. First, it may prove useful when very rapid (5 kHz to 1 MHz), prolonged data acquisition is required, but a direct memory access (DMA) port in the host computer is not available. This is a common occurrence, especially in small- or medium-size computer systems in which DMA is reserved for disk I/O, or when software complexity may be prohibitive. Second, the memory module approach is also applicable when the amount of data to be acquired exceeds the memory capacity of the host computer. The memory module described here contains 64 kilobytes but can be upgraded to 256K with minimum effort. Third, the memory module should prove beneficial when the host computer is responsible for real-time control and cannot tend to data acquisition responsibilities. Because the memory unit can be built for modest cost (less than $500) it may be more economically feasible than an additional computer system to collect data.

### References

1. Pardue, H. L., in *Topics in Automatic Chemical Analysis*; Ed. Foreman, J. K., and Stockwell, P. B. (Horwood, Chichester, 1982), pp. 163–207.
2. Murray, R. W., Heineman, W. R. and O'Dom, G. W., *Analytical Chemistry*, **39** (1981), 1666.
3. McCreery, R. L., *Analytical Chemistry*, **49** (1977), 206.
4. Rossi, D. T. and Pardue, H. L., *Analytica Chimica Acta*, **155** (1983), 103.
5. Evans, M. and Carnalli, C., *Electronic Design*, **30** (1982), 119.
6. Kuwana, T. and Strojek, J. W., *Discussions Faraday Society*. **45** (1968), 134.

